# Validation and correction of Zn–Cys*_x_*His*_y_* complexes

**DOI:** 10.1107/S2059798316013036

**Published:** 2016-09-15

**Authors:** Wouter G. Touw, Bart van Beusekom, Jochem M. G. Evers, Gert Vriend, Robbie P. Joosten

**Affiliations:** a Centre for Molecular and Biomolecular Informatics, Radboud University Medical Center, Geert Grooteplein-Zuid 26-28, 6525 GA Nijmegen, The Netherlands; b Department of Biochemistry, Netherlands Cancer Institute, Plesmanlaan 121, 1066 CX Amsterdam, The Netherlands

**Keywords:** protein zinc-binding site, zinc metal-site geometry, validation, refinement, geometric restraints

## Abstract

Many crystal structures in the Protein Data Bank contain zinc ions in a geometrically distorted tetrahedral complex with four Cys and/or His ligands. A method is presented to automatically validate and correct these zinc complexes. Analysis of the corrected zinc complexes shows that the average Zn–Cys distances and Cys–Zn–Cys angles are a function of the number of cysteines and histidines involved. The observed trends can be used to develop more context-sensitive targets for model validation and refinement.

## Abstract 2

A method is presented to automatically validate and correct Zn–Cys_*x*_His_*y*_ complexes that have a distorted tetrahedral geometry.

## 1. Introduction   

Many efforts have been directed towards improving the identification of ion types in macromolecular structures (see, for example, Sodhi *et al.*, 2004 ▸; Hsin *et al.*, 2008 ▸; Andreini *et al.*, 2009 ▸, 2013 ▸; Hemavathi *et al.*, 2010 ▸; Brylinski & Skolnick, 2011 ▸; Echols *et al.*, 2014 ▸; Zheng *et al.*, 2014 ▸; He *et al.*, 2015 ▸; Morshed *et al.*, 2015 ▸). The geometry of ion-binding sites often needs to be improved as well. The bond-valence method (Brown & Altermatt, 1985 ▸; Brese & O’Keeffe, 1991 ▸; Brown, 2009 ▸) that is generally used to identify ion types (Hooft, Vriend *et al.*, 1996 ▸; Nayal & Di Cera, 1996 ▸; Müller *et al.*, 2003 ▸; Zheng *et al.*, 2014 ▸) requires that the modelled geometry of the binding site accurately represents the crystallographic data.

Zinc ions (Zn^2+^) are the most common transition-metal ions in protein crystal structures in the Protein Data Bank (PDB; Berman *et al.*, 2007 ▸; Gutmanas *et al.*, 2014 ▸) and are the second most common metal ions overall after magnesium. Zn^2+^ ions can play a largely catalytic role or a largely structural role in proteins (see, for example, Alberts *et al.*, 1998 ▸; Lee & Lim, 2008 ▸; Sousa *et al.*, 2009 ▸; Laitaoja *et al.*, 2013 ▸), but they are sometimes also found to have nonbiological functions as crystal-packing mediators. The zinc finger is the most commonly observed zinc-binding motif in the PDB (Krishna *et al.*, 2003 ▸). It is present in protein domains with diverse functions such as binding DNA, RNA, proteins or lipids (Laity *et al.*, 2001 ▸).

Structural zinc sites typically consist of four Cys and/or His ligands (see, for example, Torrance *et al.*, 2008 ▸; Laitaoja *et al.*, 2013 ▸; Daniel & Farrell, 2014 ▸) that coordinate Zn^2+^ in a tetrahedral fashion (see, for example, Simonson & Calimet, 2002 ▸; Dudev & Lim, 2003 ▸; Lee & Lim, 2008 ▸; Torrance *et al.*, 2008 ▸). Cysteines that coordinate Zn^2+^ tend to be deprotonated (Dudev & Lim, 2002 ▸; Simonson & Calimet, 2002 ▸) and are often stabilized by hydrogen bonds to backbone H^N^ protons (Maynard & Covell, 2001 ▸). In some protein families anionic zinc environments are stabilized by the positive charges of arginine and lysine (Maynard & Covell, 2001 ▸).

Several studies have reported on the Zn^2+^—S and Zn^2+^—N distances observed in crystal structures in the PDB or the Cambridge Structural Database (CSD; Groom & Allen, 2014 ▸). These studies, summarized in Supplementary Table S1, indicate that Zn^2+^-coordination geometries are rather complex and depend, for example, on the combination of ligand types (see, for example, Simonson & Calimet, 2002 ▸; Daniel & Farrell, 2014 ▸). The stereochemical restraint targets that are commonly used to refine Zn^2+^ complexes, however, still tend to be simple and undifferentiated.

We recently reported on the inaccuracies and severely distorted geometries observed in crystallographic structure models in the PDB around tetrahedral complexes in which Zn^2+^ is coordinated by four cysteines (Evers *et al.*, 2015 ▸), and the impossible chemistry that one could naively derive from such distorted complexes was described. Although the article was published in jest on April 1st, the underlying problem we described was rather serious. Many Zn^2+^ sites in the PDB poorly describe the experimental data and show structural features that are not supported by known chemistry. This can lead to misinterpretation of the protein and incorrect answers to biological questions (Touw *et al.*, 2016 ▸).

It is easy to accidentally introduce errors during the model building and refinement of zinc sites because the use of geometric restraints between Zn^2+^ and the coordinating amino acids is not yet the default in today’s refinement programs, which, of course, is especially a problem at low resolution. The *PDB_REDO* databank (Joosten & Vriend, 2007 ▸) contained several entries in which distorted Zn^2+^ sites were accidentally introduced. Automatic detection of disulfide bonds can draw two Zn^2+^-binding cysteine side chains into a cysteine bridge, leading to the aforementioned impossible chemistry. There is currently no systematic validation of distorted metal-binding sites in the PDB validation pipeline (Read *et al.*, 2011 ▸; Gore *et al.*, 2012 ▸), which leaves distorted Zn^2+^ sites mostly undetected.

We present a method to validate Zn^2+^ complexed by cysteine and histidine ligands. The validation is based on parameters that characterize the geometry of zinc complexes and is available at the *WHAT IF* (Vriend, 1990 ▸) web server and through *WHAT_CHECK* (Hooft, Vriend *et al.*, 1996 ▸). A method to improve the geometry of zinc complexes by re-refinement, and side-chain rebuilding if required, has been implemented in *PDB_REDO* (Joosten, Salzemann *et al.*, 2009 ▸) and was applied to all PDB entries with Zn–Cys_*x*_His_*y*_ sites.

In the resulting structure models, it was observed that the ideal ion–ligand distance is not a constant, but rather a function of at least the chemical identity of the other ligands. The ideal Zn^2+^—S^γ^ distance, for example, shortens when more of the ligands are histidines (and thus fewer are cysteines). The ideal S^γ^—Zn^2+^—S^γ^ angle widens when more cysteines are replaced by histidines. These observations confirm, in protein structure models, the observations made by Simonson & Calimet (2002 ▸; Supplementary Table S1) on small-molecule data and provide a starting point from which more sophisticated, context-specific, geometric restraints for Zn^2+^-coordination sites can be developed.

## 2. Methods   

### 2.1. Geometric restraint generation   

The present study considered Cys or His side chains coordinating zinc in a tetrahedral fashion. These zinc-binding sites will be referred to as ZnCys_*x*_His_*y*_, with *x* and *y* in {0, 1, 2, 3, 4} and *x* + *y* = 4. The ligand atoms are S^γ^ for Cys and either N^δ1^ or N^∊2^ for His. For brevity, the latter two will be referred to as N^δ^ or N^∊^, respectively. The Zn^2+^ double positive charge will be implicit in notations such as Zn—N^∊^. With tetrahedral complexes we mean the collection of both tetrahedral and nearly tetrahedral complexes.

An automated method to properly refine metal complexes ideally includes the identification of the ion, the ligands and the preferred coordination number and geometric arrangement. The program *Zen* was created to perform all of the tasks necessary for preparing refinement scripts and parameters. *Zen* identifies putative ZnCys_*x*_His_*y*_ complexes in PDB entries and assumes that the ion is indeed Zn and that the ligands are arranged tetrahedrally. The reader is referred to *WHAT_CHECK* (Hooft, Vriend *et al.*, 1996 ▸) or *CheckMyMetal* (Zheng *et al.*, 2014 ▸) for validating the identity of ions when the ligands are not S^γ^, N^δ^ or N^∊^ atoms.

*Zen* searches around Zn for S^γ^ atoms within 4.8 Å and N^δ^/N^∊^ atoms within 3.8 Å. Dixon’s Q-test (Dean & Dixon, 1951 ▸) is performed on the Zn–ligand distances when five or more potential coordinating atoms are found. If four ligands are left after outlier rejection, they are assumed to constitute a ZnCys_*x*_His_*y*_ site. Complexes are discarded if (i) a different type of ligand (neither Cys S^γ^ nor His N^δ^/N^∊^) is found close to Zn (2.9 Å or closer) and (ii) a S^γ^/N^δ^/N^∊^ ligand is found 3.25 Å or further away from Zn. In order to prevent the detection of octahedral Zn sites, such as the Zn site observed in the polyketide cyclase RemF (PDB entry 3ht2; Silvennoinen *et al.*, 2009 ▸), ZnHis_4_ complexes are also discarded if only requirement (i) is satisfied. Additionally, all sites with at least three His ligands require all ligand atoms to be present within 3.0 Å of Zn. Clusters of tetrahedral Zn complexes in which individual S^γ^ atoms coordinate more than one Zn ion are also detected by *Zen*. The abovementioned distance cutoffs were optimized empirically to minimize the number of false positives (for example ZnHis_6_ sites detected as ZnHis_4_ sites) and false negatives (undetected ZnCys_*x*_His_*y*_ sites).

The fact that many PDB file headers have missing or spurious LINK records for distorted sites as well as SSBOND records between cysteines coordinating a zinc ion (Evers *et al.*, 2015 ▸) poses a problem for the refinement program *REFMAC* (Murshudov *et al.*, 2011 ▸) which is used in *PDB_REDO*. Incorrect annotation of the covalent and metal-coordination bonds causes *REFMAC* to generate incorrect geometry restraints. The authors have contacted the developers of *REFMAC* to prevent the activation of cysteine-bridge restraints when at least one of the cysteines is also involved in a zinc-coordination LINK record. The annotation of ZnCys_*x*_His_*y*_ complexes, however, still has to be correct and complete to prevent refinement problems. Therefore, all SSBOND and LINK records involving ZnCys_*x*_His_*y*_ complexes are corrected by *Zen*, resulting in so-called Cys-cleaned PDB files.

Based on the re-annotated LINK records, *REFMAC* imposes distance and angle restraints during refinement. The distance-restraint targets presently are 2.340 ± 0.020 Å for Zn—S^γ^, 2.057 ± 0.064 Å for Zn—N^δ^ and 2.058 ± 0.073 Å for Zn—N^∊^. Zn—S^γ^—C^β^ angles are restrained to 109.000 ± 3.000°. Zn—N^δ^—C^γ^, Zn—N^δ^—C^∊^, Zn—N^∊^—C^δ^ and Zn—N^∊^—C^∊^ angles are restrained to 125.350 ± 3.000°. The Zn–Cys distance and angle targets were already present in the *REFMAC* dictionary (Vagin *et al.*, 2004 ▸). The Zn–His distance targets were obtained from tetrahedral complexes in the MESPEUS database (Hsin *et al.*, 2008 ▸) solved at 1.6 Å resolution or better and were added to the *REFMAC* refinement dictionary. The associated Zn—N^δ^—C^γ^, Zn—N^δ^—C^∊^, Zn—N^∊^—C^δ^ and Zn—N^∊^—C^∊^ angle targets were set to the same as the values for the H^∊2^ and H^δ1^ atoms. The numeric precision in the new restraints described above is kept consistent with the existing restraints, but the significant digits do not represent the accuracy at which bond angles are determined.

The *REFMAC* dictionary currently does not provide a mechanism to add angle restraints that involve three separate compounds (*i.e.* the Zn and two coordinating residues). Therefore, the (ligand 1)–Zn–(ligand 2) angles cannot be restrained automatically. The absence of these restraints allows Zn sites to depart from tetrahedral geometry without severely violating the available geometric restraints. Additionally, without these restraints it is difficult to recover, by refinement only, from the distorted geometries that we have described previously (Evers *et al.*, 2015 ▸). *Zen* therefore creates specific angle restraints that can be applied in refinement using the external restraints mechanism in *REFMAC* (Nicholls *et al.*, 2012 ▸). The target for S^γ^—Zn—S^γ^ angles was set to the ideal tetrahedral value of 109.5 ± 3.0°. Angles involving histidine are not restrained because the position of histidine side chains in Zn sites is much better defined than those of cysteine side chains because of the size and rigidity of the imidazole group.

### 2.2. Updates to *PDB_REDO*   

The *PDB_REDO* pipeline (Joosten, Salzemann *et al.*, 2009 ▸) was extended to include the refinement of ZnCys_*x*_His_*y*_ complexes. In the initial stage, *Zen* is run when a model contains at least one Zn ion. The *PDB_REDO* program *extractor* (Joosten, Womack *et al.*, 2009 ▸) was updated to add Zn ions to the TLS (Schomaker & Trueblood, 1968 ▸) group of the coordinating residues, provided that they are all part of the same macromolecular chain. This applies only to the TLS-group selections created by *extractor*; TLS-group selections provided by the user or extracted from the header of the PDB file are purposely left unchanged. During the initial re-refinement with *REFMAC*, the external restraints generated by *Zen* are applied with default weights. For the sake of this study, automated disulfide-bond detection in *REFMAC* was switched off to prevent *REFMAC* from generating erroneous disulfide-bond restraints when cysteine side chains are too close. As a result of our findings, *REFMAC* was updated to not generate disulfide-bond restraints if one of the cysteine S^γ^ atoms is involved in a LINK record. Automated cysteine-bridge detection in *REFMAC* is therefore switched back on again in the latest version of *PDB_REDO*.

Re-refinement and subsequent model rebuilding (Joosten *et al.*, 2011 ▸) can change the structure model to such an extent that previously undetected ZnCys_*x*_His_*y*_ complexes can be identified. If this is the case, *Zen* updates the model annotation and external restraints and the second round of model refinement is extended to increase the probability of convergence. For example, the ZnCys_4_ complex around Zn A2456 in RNA polymerase II in PDB entry 2b63 (Kettenberger *et al.*, 2006 ▸) is not detected because the Zn—S^γ^ distance for Cys107 is above the detection threshold (5.70 Å). After re-refinement the distance is just below (4.73 Å) the detection threshold. Consequently, the ZnCys_4_ complex is recognized by *Zen* and during a second round of refinement the distance decreases to 2.35 Å.

The updated *PDB_REDO* pipeline was used to replace all entries of the *PDB_REDO* databank (Joosten & Vriend, 2007 ▸) containing ZnCys_*x*_His_*y*_ sites.

### 2.3. ZnCys_*x*_His_*y*_ geometry validation   

Features characterizing the ZnCys_*x*_His_*y*_ coordination complexes were determined using *WHAT IF* (Vriend, 1990 ▸). These features included bond distances, angles, torsion angles, point charge distributions, the presence and apparent multiplicity of cysteine bridges, the Zn position in the tetrahedron, and atom occupancies and *B* factors. His side-chain flips (Hooft, Sander *et al.*, 1996 ▸) and crystallographic symmetry (Hooft *et al.*, 1994 ▸) can be taken into account by the validation routines. The sample mean and standard deviation of each feature were determined as a function of the ligand composition. In order to prevent bias from different refinement strategies, these statistics were not derived from original sites but from sites that had been re-refined with *PDB_REDO* using the abovementioned undifferentiated restraint targets. *Z*-scores were calculated for the distances, angles and Zn position in the tetrahedron because manual inspection showed that these features were most indicative of the quality of the ZnCys_*x*_His_*y*_ complex. A combined quality metric was constructed by calculating the root-mean-square *Z*-score (r.m.s.*Z*). The optimal value of an r.m.s.*Z* statistic varies between 0.0 at low resolution and 1.0 at high resolution (Tickle, 2007 ▸).

## 3. Results   

### 3.1. The geometric quality of ZnCys_*x*_His_*y*_ complexes is improved   

**Figure 1:**
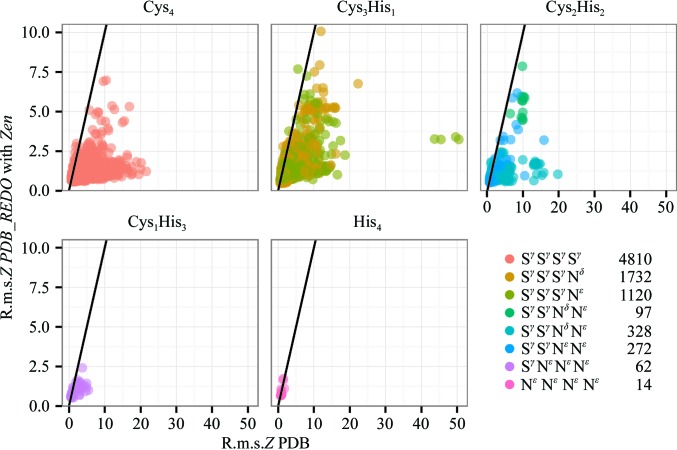
R.m.s.*Z* for the five possible ZnCys_*x*_His_*y*_ site types. The scales on the two axes are different; black lines indicate the situation where the r.m.s.*Z* is the same for complexes in the PDB and after *Zen* remediation and re-refinement in *PDB_REDO*. Ligand atoms and site counts are indicated in the legend.

8610 ZnCys_*x*_His_*y*_ complexes were detected in 3110 PDB entries (April 20th 2016) and subjected to optimization by *PDB_REDO* with and without *Zen* remediation. The validation routines detected that 170 sites contained Zn ligands next to a chain break and that five PDB complexes [in PDB entries 4hoo (Krishnan & Trievel, 2013 ▸), 4tvr (Structural Genomics Consortium, unpublished work) and 5etx (Soumana *et al.*, 2016 ▸)] contained incompletely built Zn ligands that had been completed by *PDB_REDO*. These outliers were removed from the subsequent analyses. The 8435 tetrahedral ZnCys_*x*_His_*y*_ complexes resulted in nearly all cases in a higher overall tetrahedral coordination geometry quality after processing by *Zen* and optimization by *PDB_REDO* (Fig. 1 ▸ and Supplementary Fig. S1). The average r.m.s.*Z* was 2.65 ± 9.89 for PDB complexes, 1.78 ± 2.07 after optimization without *Zen* remediation and 1.14 ± 0.60 after optimization with *Zen* remediation. The median r.m.s.*Z* was 1.58, 1.15 and 1.00, respectively. A median decrease of 5.59 was observed for the 10% most improved complexes. 217 complexes had an r.m.s.*Z* that was above 1.00 in the PDB (average 1.33 ± 0.43, median 1.20) and lower than the r.m.s.*Z* after *Zen* remediation (average 1.49 ± 0.60, median 1.33). Only 58 complexes had an r.m.s.*Z* below 1.00 (0.91 ± 0.06) in the PDB and above 1.00 in *PDB_REDO* (1.10 ± 0.10). In line with our treatment of bond-length and bond-angle r.m.s.*Z* scores on the *PDB_REDO* server (Joosten *et al.*, 2014 ▸), we regard these 275 complexes (3.3% of the total number of complexes) as deteriorated.

**Figure 2:**
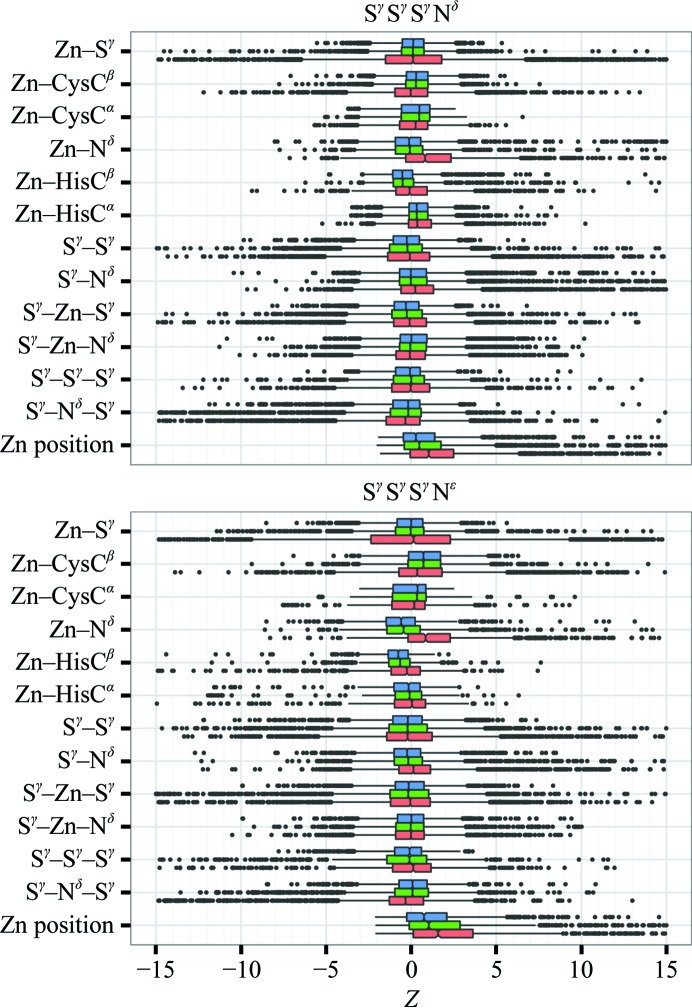
Box-and-whisker plots of the *Z*-scores characterizing ZnCys_3_His_1_ complexes in *PDB_REDO* with *Zen* remediation (blue), *PDB_REDO* without *Zen* remediation (green) and original PDB (red) structure models. The whiskers extend to the nearest value that is within 1.5 times the inter-quartile range; outliers are marked as dots. The *Z* score for ‘Zn position’ indicates the deviation from the expected Zn position in the tetrahedron. 1411 outliers with a *Z*-score outside (−15, +15) are not shown for clarity. 891 of these outliers are from PDB structure models, while 476 and 44 outliers are from *PDB_REDO* entries without and with *Zen* remediation, respectively.

Generally, the individual *Z*-score components of r.m.s.*Z* also improved. *PDB_REDO* models after *Zen* remediation have *Z*-score distributions that cluster more tightly around the expected values and have fewer outliers than PDB models (to a smaller extent this is also observed for *PDB_REDO* models that have not been processed by *Zen*). This is exemplified for the features capturing the geometric quality of ZnCys_3_His_1_ complexes in Fig. 2 ▸. As expected, parameters that were directly targeted because they had been restrained (*e.g.* Zn—S^γ^, Zn—N^δ^ and Zn—N^∊^ distances and S^γ^—Zn—S^γ^ angles) or Cys-cleaned (S^γ^—S^γ^ distances) on average improved most. Notably, the Zn—S^γ^ *Z*-score distribution is essentially symmetric in the PDB, *i.e.* Zn—S^γ^ distances are either too long or too short, whereas Zn—N^δ^ or Zn—N^∊^ distances in the PDB are typically too long. This may be caused by the absence of a standard target in the restraint dictionaries, but, at least for structure models refined by *REFMAC*, also by the presence of ‘riding’ H atoms on the N^δ^ or N^∊^ atoms during refinement in the absence of LINK records (that describe a bond-length target plus the explicit deprotonation of these N atoms). These H atoms push the Zn ions and the histidine N atoms apart. The median *PDB_REDO* ZnCys_3_His_1_ Zn—N distance is smaller than expected, most likely because the undifferentiated restraint target distances (see §[Sec sec2]2) are much shorter than the ZnCys_3_His_1_-specific validation targets: at 1.6 Å resolution the average overall Zn—N distance is 2.074 ± 0.056 (see below). On a more detailed level, Zn—N^δ^ distances are 2.076 ± 0.057 and Zn—N^∊^ distances are 2.065 ± 0.050 on average. Zn—C^β^ distances are not directly restrained (although Zn—C^β^ distances are influenced by Zn—S^γ^—C^β^ angle restraints) and their median deviates more from the expected values in *PDB_REDO* complexes than in PDB complexes. The number of Zn—C^β^ distance outliers in *PDB_REDO* complexes is reduced at the same time.

The changes in geometric parameters for the other four ZnCys_*x*_His_*y*_ complexes are shown in Supplementary Fig. S2 and follow similar patterns.

**Figure 3:**
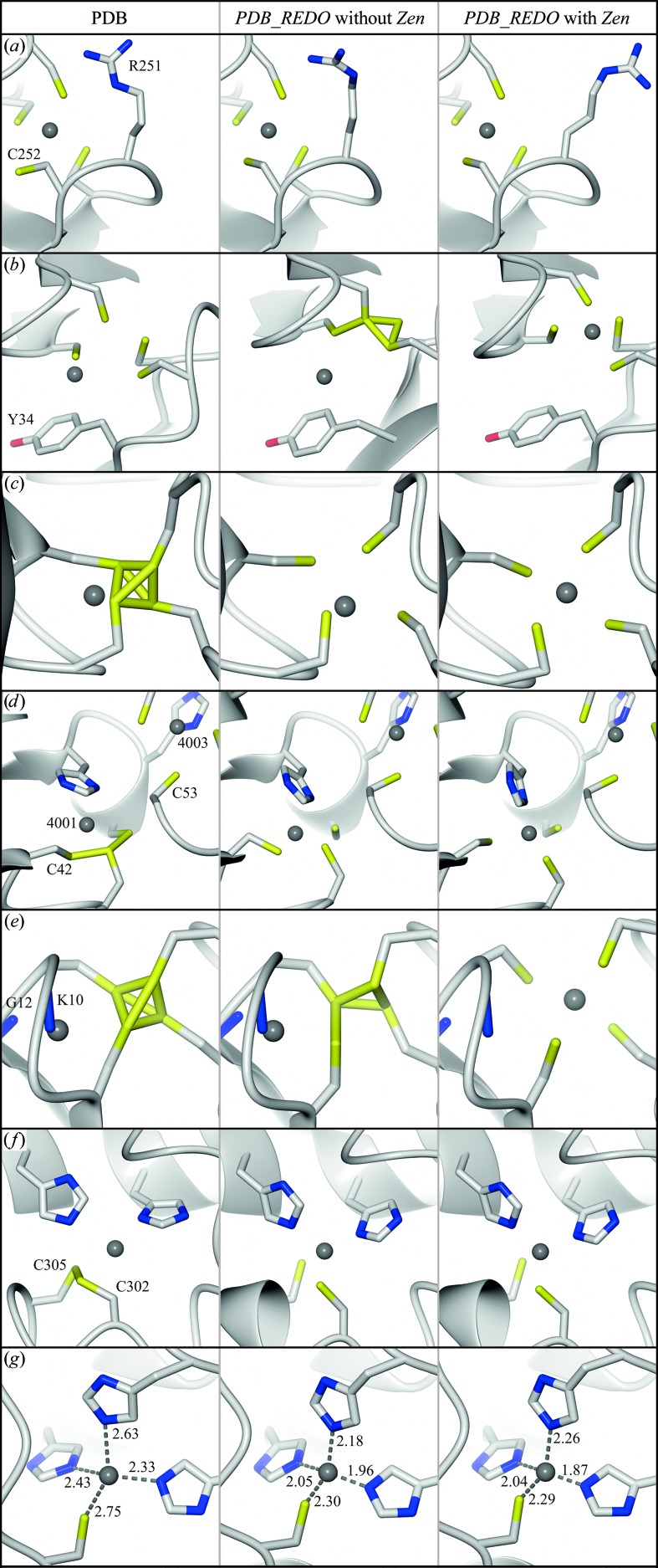
ZnCys_*x*_His_*y*_ complexes before (left) and after *PDB_REDO* without (middle) and with (right) *Zen* remediation. Side chains are coloured by atom type; grey spheres are Zn ions. Figures were prepared with *CCP*4*mg* (McNicholas *et al.*, 2011 ▸). Electron-density maps were omitted for clarity and are available from the *PDB_REDO* databank. (*a*) Zn300, chain *A*, from the 8-oxoguanine DNA glycosylase MutM (PDB entry 1l1z; 1.7 Å; Fromme & Verdine, 2002 ▸). Cys252 points away from the Zn ion. The LINK between Cys252 and Zn was not annotated in the PDB model. In the *PDB_REDO* models Cys252 S^γ^ has moved 2.7 Å. Arg251 was refitted to a more plausible conformation only after *Zen* detected the ZnCys_4_ site. (*b*) Zn203, chain *I*, from the RNA polymerase II–transcription factor IIB complex (PDB entry 1r5u; 4.5 Å; Bushnell *et al.*, 2004 ▸). Zn203 is modelled far away from the centre of the four S^γ^ ligands. The presence of a LINK record between Zn and C^δ2^ of Tyr34 and the absence of three S^γ^—Zn LINK records in the PDB file precludes complex formation in a standard (re-)refinement. Correction of the Zn site required the Zn to move more than 5 Å. (*c*) Zn313, chain *B*, from aspartate transcarbamoylase (PDB entry 3d7s; 2.8 Å; Stieglitz *et al.*, 2009 ▸). Several types of cysteine-bridge problems exist in the PDB (Evers *et al.*, 2015 ▸), and the four cysteines next to Zn313 form an extreme example. Only three of the four necessary LINK records are specified in the original PDB file and at the same time superfluous SSBOND records are present for three of the six bridges shown. The cysteine clashes are almost resolved even without *Zen* processing thanks to the adaptations that were made to *REFMAC* as a result of our work. The additional restraints generated by *Zen* were necessary to refine the Zn position correctly. (*d*) Zn4001, chain *D*, from the DDB1–Cul4A–Rbx1–SV5V complex (PDB entry 2hye; 3.1 Å; Angers *et al.*, 2006 ▸). The three cysteines and the histidine are not arranged tetrahedrally around Zn4001 and the three cysteines appear to form one big cysteine bridge. Without *Zen* remediation the r.m.s.*Z* is 9.69. The correct Cys42 rotamer was found during re-refinement after processing with *Zen*, allowing better refinement of the Zn and ligand positions (final r.m.s.*Z* of 1.09). The Zn4003 site is located close to the Zn4001 site and has a tetrahedral conformation. In the PDB entry the distance from the C^β^ atom of Cys53 to Zn4001 is 4.38 Å, whereas the distance to Zn4003 is 4.20 Å. *Zen* detected correctly that Cys53 only coordinates Zn4003. (*e*) Zn61, chain *B*, from the box H/ACA ribonucleoprotein protein particle–RNA complex (PDB entry 3lwq; 2.7 Å; Zhou *et al.*, 2010 ▸). Four cysteines are tightly connected near the Zn. In the PDB entry SSBOND records are present for these cysteines, while LINK records for the Zn are found to the backbone N atoms of Gly12 and Lys10. Normal ZnCys_4_ geometry is obtained in the *Zen*-processed *PDB_REDO* model. The ion has moved 3.5 Å. (*f*) Zn6, chain *C*, of the *Simian virus 40* large T-antigen–human p53 complex (PDB entry 2h1l; 3.2 Å; Lilyestrom *et al.*, 2006 ▸). For 12 of the 24 chains in the PDB model SSBOND records are specified between Cys302 and Cys305, while these two residues actually coordinate the Zn together with two histidines. The complex was refined correctly with and without processing by *Zen*. (*g*) Zn4, chain *B*, from the catalytic domain of human AMSH (PDB entry 3rzu; 2.5 Å; Davies *et al.*, 2011 ▸). The coordination distances are too large. The distances in the *PDB_REDO* models were closer to the expected values.

Visual inspection showed that a lower r.m.s.*Z* corresponds to a more plausible geometry and that most of the severely distorted ZnCys_*x*_His_*y*_ complexes improved dramatically upon re-refinement. Special, complicated cases such as the Cys_3_–Zn–Cys_1_–Zn–Cys_2_His_1_ complex in the UBR box of E3 ubiquitin ligase (PDB entry 3nih; Choi *et al.*, 2010 ▸) and the ZnCys_4_ site between the two Get3 chains in the Get3–Get1 complex (PDB entry 3sjb; Stefer *et al.*, 2011 ▸) were handled correctly by our method. Fig. 3 ▸ shows several examples of complex problems that were solved satisfactorily.

Taken together, it was observed that *PDB_REDO* optimization without *Zen* remediation leads to a tighter distribution of geometry scores and that the extra *Zen* processing step further improves the average geometric quality by removing additional outliers (without significantly changing the average *B* factor; see Supplementary Fig. S3). Supplementary Fig. S4 shows examples of the classes of outliers that were still observed in our data set. These challenges include false-positive detection of ZnCys_*x*_His_*y*_ complexes when one of the true Zn ligands is not Cys or His (Supplementary Fig. S4*a*), spurious LINKs between Zn ligands ( Supplementary Fig. S4*b*; most of these problems have been resolved in the most recent version of *Zen*) and undetected His side-chain flips (Supplementary Fig. S4*c*).

The fully automated detection of missing waters is a longstanding problem in crystallography and is particularly challenging in the vicinity of metal ions (Supplementary Fig. S5).

### 3.2. ZnCys_*x*_His_*y*_ refinement targets are context-dependent   

**Figure 4:**
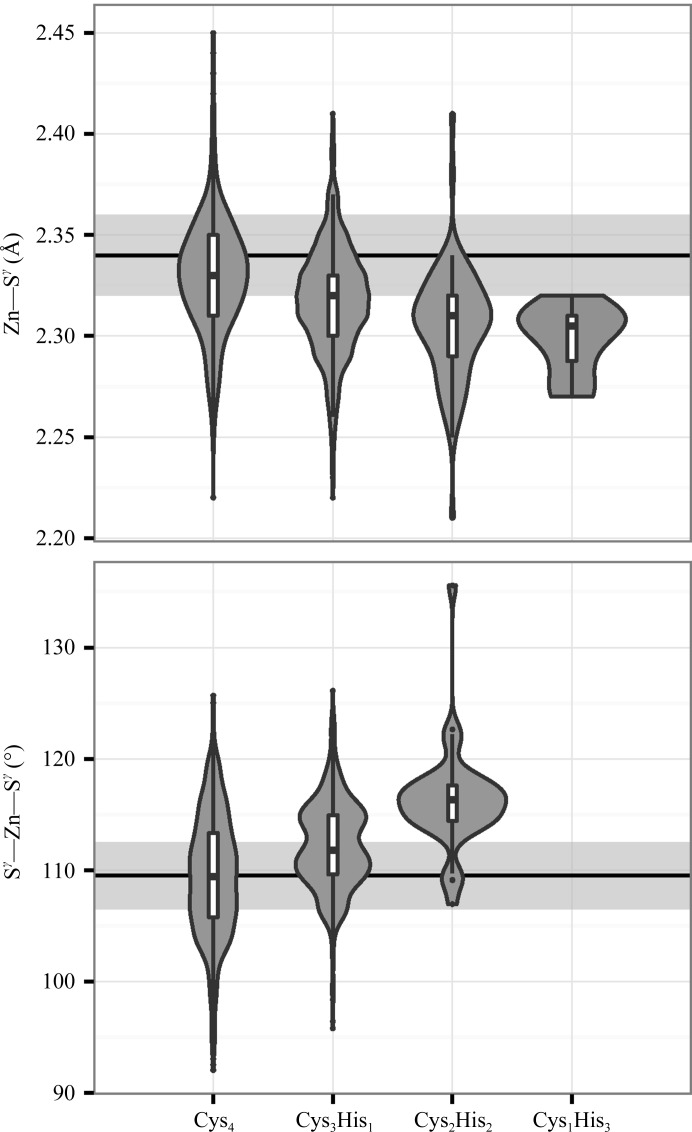
Zn—S^γ^ distance (top) and S^γ^—Zn—S^γ^ angle (bottom) distributions as a function of the number of cysteines and histidines in ZnCys_*x*_His_*y*_ complexes determined at 1.6 Å resolution or better. The contours of the violin plots are kernel density estimates and the box plots are shown as in Fig. 2 ▸. The light grey background areas show one standard deviation around the refinement targets for the Zn—S^γ^ distance (2.340 ± 0.020 Å) and the S^γ^—Zn—S^γ^ angle (109.5 ± 3.0°). The difference between the types of ZnCys_*x*_His_*y*_ complexes is significant (see Table 1 ▸). When Zn is coordinated by N^δ^ in ZnCys_3_His_1_ complexes, the S^γ^—Zn—S^γ^ angle distribution is somewhat bimodal and partly depends on the rotameric state and backbone conformation of the cysteines.

The Zn—S^γ^ distances and S^γ^—Zn—S^γ^ angles were calculated as a function of ligand identity for the set of re-refined complexes from which 5σ outliers were iteratively removed. Fig. 4 ▸ shows that the refined distances and angles are different from their refinement targets and that the refined distances and angles are not constant but are a function of the ligand composition of the ZnCys_*x*_His_*y*_ complex.

## 4. Discussion   

### 4.1. Automated restraint generation   

The feasibility of fully automatically generating refinement restraints for metal sites depends on the quality of the structure model and the prior knowledge of the correct geometry. The effect of errors in the atomic coordinates on structural interpretation of a metal site for restraint generation is less severe if accurate prior knowledge is available from other experiments or data mining. Here, we show that effective restraints can be generated for Zn sites with predicted tetrahedral geometry, even when the input model is severely distorted. ZnCys_*x*_His_*y*_ complexes have better r.m.s.*Z* scores after optimization by *Zen* and *PDB_REDO*. These scores are a combined measure of geometric variables in the context of an entire ZnCys_*x*_His_*y*_ complex. The *Z*-score distributions seem to indicate that the total quality sometimes improves at the cost of a worse score for an individual r.m.s.*Z* component. This might for example be caused by incorrect restraint targets (see below), the effect of which is only problematic at low resolution, or, more generally, by difficulty in escaping local refinement minima. At the same time, however, the number of outliers decreased for all geometric variables.

If not all Zn ligands are modelled, the site will remain undetected and no restraints are generated. For catalytic Zn sites it is difficult to predict the geometry, and restraints must be made manually. Alternatively, refinement can be performed using computationally more expensive methods based on quantum mechanics (QM), such as the semi-empirical QM refinement in *PHENIX*/*DivCon* (Borbulevych *et al.*, 2014 ▸). Metal sites may be refined without restraints when crystallographic data are of sufficient quality and resolution.

The methods developed here can, when sufficient examples are available in the PDB, be extended to other ligand compositions of tetrahedral zinc complexes, *e.g.* Zn sites that involve water, but also to other geometries and other ion types, such as octahedral magnesium sites that are often observed in nucleic acid structures.

### 4.2. Validation using electron density   

**Figure 5:**
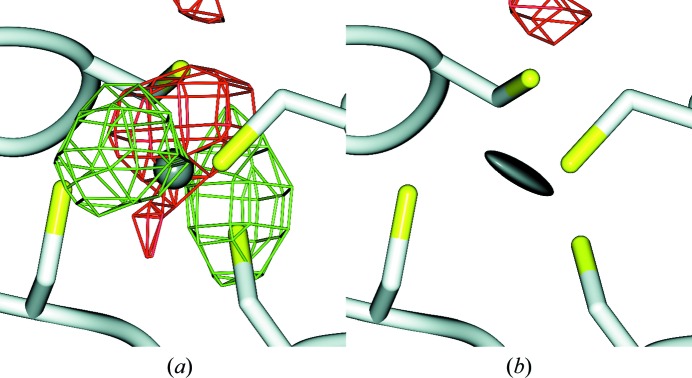
Zn1702, chain *B*, from jumonji H3K27 demethylase (PDB entry 4eyu; Kruidenier *et al.*, 2012 ▸). *mF*_o_ − *DF*_c_ difference electron-density maps after a *PDB_REDO* run with (*a*) an isotropic *B* factor for Zn^2+^ (grey sphere) or (*b*) an anisotropic *B* factor for Zn^2+^ (grey thermal ellipsoid). The maps (positive, green mesh; negative, red mesh) are contoured at 3σ, are rendered with a grid size of 0.77 Å and for clarity are shown only in the vicinity of the Zn. The largest atomic displacement between any atom in this ZnCys_4_ complex between (*a*) and (*b*) is 0.16 Å.

Improvement of a crystallographic structure model generally leads to an improvement of the corresponding electron-density map (EDM). The real-space correlation coefficient (RSCC) measures the fit of the atoms to the EDM, but correlates strongly with metrics of model precision such as the atomic *B* factors (Tickle, 2012 ▸). Particularly at low resolution, the RSCC metric becomes less reliable. Tickle (2012 ▸) suggested the real-space difference density *Z*-score (RSZD) as an EDM metric that only correlates with model accuracy and not with model precision. We did not observe a clear correlation between the geometric quality of ZnCys_*x*_His_*y*_ complexes and their fit to the EDM measured by either the RSCC or RSZD. It was observed that a complex can have reasonable EDM metrics even when it is very bad in terms of geometry, and *vice versa*. In our hands these EDM metrics therefore were not very helpful in determining whether re-refinement of ZnCys_*x*_His_*y*_ complexes was successful or not. The validation was therefore solely based on geometric parameters. We did observe in many cases, though, that re-refinement with inclusion of anisotropy for just the Zn ions led to visually more pleasing EDMs with less difference density around the Zn (see Fig. 5 ▸ for an example). Anisotropic atomic displacement can be partially modelled using the TLS formalism and this is currently implemented in *PDB_REDO*. Zn and other heavy atoms may be refined with anisotropic *B* factors systematically in a future implementation, provided that the data-to-parameter ratio is not severely affected. This implementation may also need to include and optimize *B*-factor sphericity restraints in order to balance residual difference density and *B*-factor anisotropy.

### 4.3. Context-specific refinement targets   

The original Engh and Huber parameters (Engh & Huber, 1991 ▸, 2001 ▸) are targets for bond lengths and angles and are averages for all conceivable situations. The very large number of high-resolution structures available from the PDB today allows fine-detailing of these parameters, as has, for example, been shown in a study on the angle τ, the N—C^α^—C angle (Touw & Vriend, 2010 ▸). This large volume of data allows us to start determining better parameters for restraints for distances and angles in ZnCys_*x*_His_*y*_ complexes. Clearly, these parameters are also determined by the local environment. For example, the Zn—S^γ^ distance is shorter when the number of coordinating cysteines is smaller. QM calculations have suggested that this trend partly correlates with a smaller electrostatic repulsion between the thiolate S atoms and that steric and stabilizing electrostatic interactions from the secondary coordination sphere have an effect on zinc-site geometry (Simonson & Calimet, 2002 ▸; Daniel & Farrell, 2014 ▸). These findings imply that further fine-detailing will be possible as a function of the presence of nearby positive or negative groups. We indeed observe an excess of positively charged amino acids close to many, but not all, ZnCys_*x*_His_*y*_ complexes. Counting statistics presently still preclude taking such details into account. Only when more data become available, especially at high resolution, will we be able to express target values as a function of more environmental factors and determine which environmental factors influence the target values most. The Zn—S^γ^, S^γ^—Zn—S^γ^, Zn—N and N—Zn—N parameters for tetrahedral ZnCys_*x*_His_*y*_ complexes that we observe in the *PDB_REDO* databank in the subset of structures solved at a resolution of 1.6 Å or better are listed in Table 1 ▸.

There are not yet enough data to treat N^δ^ and N^∊^ separately and there are limited data available for ZnCys_1_His_3_ and ZnHis_4_ sites. The parameters in Table 1 ▸ depend significantly on the type of ZnCys_*x*_His_*y*_ complex. However, the data show signs of an underlying multimodality that we cannot yet fully resolve (Fig. 4 ▸). Nevertheless, these parameters provide a starting point for making more sophisticated sets of restraints, and the growth of the PDB and the *PDB_REDO* databank will provide more reliable statistics over time. Like many other geometric values (see, for example, Touw & Vriend, 2010 ▸), the ZnCys_*x*_His_*y*_ values are a function of crystallographic resolution. The values that we observe for structures solved at a resolution of 2.5 Å or better (Supplementary Table S2) are slightly different from those in Table 1 ▸ but follow the trends described above.

Extracting restraints from the *PDB_REDO* databank and subsequently applying them in the *PDB_REDO* pipeline introduces circularity. This important practical issue can be avoided by only applying these restraints to low-resolution structure models (where the restraints are most needed) and not to the high-resolution structure models that will be used to derive new refinement targets. In this way, future data sets will remain unbiased. Restraint targets ideally are derived from unrestrained Zn sites, but the number of available ZnCys_*x*_His_*y*_ complexes solved at atomic resolution will preclude the extraction of statistically significant targets from unrestrained structure models for some time to come.

## 5. Conclusion   

The geometry of both moderately and severely distorted ZnCys_*x*_His_*y*_ sites in the PDB could be improved substantially by restraining the sites to tetrahedral coordination geometry using both Zn–ligand distance restraints and tetrahedral S^γ^—Zn—S^γ^ angle restraints. Correcting geometry using refinement with restraints based on prior chemical knowledge and validating the results require that accurate refinement targets are known. Geometric trends in systematically re-refined ZnCys_*x*_His_*y*_ sites show that current restraint targets may be replaced by context-specific targets. Context-specific angle restraint targets will soon be implemented in *PDB_REDO* and context-specific distance targets will follow subject to the availability of a suitable framework for these in *REFMAC*. Geometric targets for ZnCys_*x*_His_*y*_ sites may be further detailed once sufficient data are available.

## 6. Availability   

The functionality to improve the refinement of ZnCys_*x*_His_*y*_ sites is available through the *PDB_REDO* web server (Joosten *et al.*, 2014 ▸). *Zen* is distributed with *PDB_REDO* and the source code is available upon request. The *WHAT IF* web servers and web services are freely available and *WHAT IF* is shareware. *WHAT_CHECK* and *PDB_REDO* will become part of the *CCP*4 software suite (Winn *et al.*, 2011 ▸) soon. A large .csv file that contains all of the data used for analysing the 8435 tetrahedral ZnCys_*x*_His_*y*_ complexes is available as supplementary data.

## 7. Related literature   

The following references are cited in the Supporting Information for this article: Chung *et al.* (2005 ▸), Duan *et al.* (2009 ▸), Harding (2006 ▸), LaPlante *et al.* (2014 ▸), Ma *et al.* (2015 ▸), Samara *et al.* (2012 ▸) and Tamames *et al.* (2007 ▸).

## Supplementary Material

Supporting Information.. DOI: 10.1107/S2059798316013036/rr5124sup1.pdf

Bzip2-compressed CSV file with raw numerical data. DOI: 10.1107/S2059798316013036/rr5124sup2.bin

## Figures and Tables

**Table 1:** Suggested refinement targets for the five possible ZnCys_*x*_His_*y*_ complex types The targets have been derived from crystallographic structures determined at a resolution of 1.6 Å or better and are listed as mean ± standard deviation. Numbers in parentheses indicate the number of observations. For all targets a significant difference between means was observed across the types of ZnCys_*x*_His_*y*_ complexes [one-way ANOVA with a Welch correction for nonhomogeneity of variances (Welch, 1951 ▸): Zn—S^γ^ distance, *F*_(3, 49.5)_ = 50.7, *p* = 4.1 × 10^−15^; S^γ^—Zn—S^γ^ angle, *F*_(2, 100.3)_ = 124.7, *p* << 10^−15^; Zn—N distance, *F*_(2, 86.9)_ = 45.5, *p* = 3.1 × 10^−14^; N—Zn—N angle, *F*_(1, 71.6)_ = 16.6, *p* = 1.2 × 10^−4^]. The same parameters derived from crystallographic structures determined at a resolution of 2.5 Å or better are given in Supplementary Table S2.

Zn—S^γ^ (Å)	S^γ^—Zn—S^γ^ (°)	Zn—N (Å)	N—Zn—N (°)	ZnCys_*x*_His_*y*_
2.330 ± 0.029 (1033)	109.45 ± 5.46 (1553)	n/a	n/a	Cys_4_
2.318 ± 0.027 (912)	112.15 ± 3.96 (912)	2.074 ± 0.056 (303)	n/a	Cys_3_His_1_
2.306 ± 0.029 (76)	116.23 ± 4.58 (38)	2.040 ± 0.050 (65)	102.38 ± 5.44 (38)	Cys_2_His_2_
2.298 ± 0.017 (12)	n/a	2.002 ± 0.045 (36)	107.23 ± 4.78 (36)	Cys_1_His_3_
n/a	n/a	Insufficient data	Insufficient data	His_4_
