# Supplementary material for: Validation and correction of Zn–CysxHisy complexes
Source: Acta Crystallogr D Struct Biol. 2016 Sep 15;72(Pt 10):1110–8. doi: 10.1107/S2059798316013036 (PMC5053137; doi:10.1107/S2059798316013036)
Supplement: Supplementary file 1 [file d-72-01110-sup1.pdf]

STRUCTURAL BIOLOGY

Volume 72 (2016)

Supporting information for article:

Validation and correction of Zn–$Cys_x$$His_y$ complexes

Wouter G. Touw, Bart van Beusekom, Jochem M. G. Evers, Gert Vriend and Robbie P. Joosten

Supporting Information

S1. Zn—S and Zn—N distances

Supplementary Table S1. Distances between Zn and S or N are listed as mean ± standard deviation.

Study	Zn—S (Å)	Zn—N (Å)	Databank	Resolution (Å) and other criteria	Site type
**(Alberts *et al.*, 1998)**	2.35 ± 0.09 (30)	2.09 ± 0.12 (42)	PDB	≤ 2.0 Structural sites only	T4
	2.30 ± 0.05 (147)	2.02 ± 0.03 (27)	CSD	-	T4
**(Simonson & Calimet, 2002)**	2.33 ± 0.05 (60)	n/a	PDB	≤ 2.25	Cys_4
	2.34 ± 0.10 (51)	2.19 ± 0.19 (17)	PDB	≤ 2.25	Cys_3His
	2.25 ± 0.09 (28)	2.12 ± 0.18 (28)	PDB	≤ 2.25	Cys_2His_2
	2.36 ± 0.06 (44)	n/a	CSD	-	SSSS
	2.31 ± 0.03 (24)	2.12 ± 0.03 (8)	CSD	-	SSSN
	2.28 ± 0.02 (30)	2.09 ± 0.04 (30)	CSD	-	SSNN
	2.22 ± 0.08 (3)	2.06 ± 0.02 (9)	CSD	-	SNNN
**(Harding, 2006)**	2.34 ± 0.05 (59)	2.04 ± 0.04 (62)	PDB	≤ 1.25	All
	2.28 ± 0.04 (28)	2.01 ± 0.04 (34)	CSD	$R < 0.065$	All
	2.31 ± 0.10	2.03 ± 0.05	*	*	All
**(Tamames *et al.*, 2007)**	2.32 ± 0.11 (1877)	Nδ: 2.09 ± 0.14 (1115)<br>Nε: 2.12 ± 0.15 (2347)	PDB	≤ 2.5	All
	n/a (0)	2.02 ± 0.04 (11)	PDB	0.5-1.0	All
	2.33 ± 0.05 (90)	2.08 ± 0.09 (180)	PDB	1.0-1.5	All
	2.32 ± 0.09 (732)	2.10 ± 0.13 (1967)	PDB	1.5-2.0	All
	2.32 ± 0.13 (1094)	2.13 ± 0.16 (1470)	PDB	2.0-2.5	All
**(Lee & Lim, 2008)**	2.33 ± 0.09 (137)	2.05 ± 0.16 (36)	PDB	≤ 3.0 Structural sites only	T4
	2.32 ± 0.05 (246)	2.04 ± 0.04 (603)	CSD	$R < 0.065$	T4
**(Laitaoja *et al.*, 2013)**	2.31 ± 0.03 (24)	2.03 ± 0.04 (33)	PDB	<1.0	All
	2.32 ± 0.06 (436)	2.08 ± 0.08 (637)	PDB	1.0-1.5	All
	2.34 ± 0.10 (2116)	2.13 ± 0.15 (2279)	PDB	1.5-2.0	All
	2.36 ± 0.20 (1928)	2.18 ± 0.17 (1579)	PDB	2.0-2.5	All
	2.36 ± 0.23 (920)	2.25 ± 0.28 (953)	PDB	2.5-3.0	All
	2.40 ± 0.28 (428)	2.26 ± 0.23 (211)	PDB	3.0-3.5	All
	2.64 ± 0.67 (131)	2.58 ± 0.43 (19)	PDB	3.5-4.0	All
	2.45 ± 0.49 (119)	2.86 ± 1.26 (5)	PDB	>4.0	All

From left to right are listed the source reference, any Zn-S and Zn-N distances listed, the data source, resolution cut offs when the source was PDB and *R*-factor cut-offs when the source was CSD, and the type of cluster studied (with all indicating lack of discrimination, SSSS indicates the general case of Zn coordination by four sulphur atoms, and T4 indicating that the coordination number had to be 4). Numbers in brackets indicate the number of observations. *: suggested targets by Harding (2006).

S1.2 Targets observed in this study

Targets observed at a resolution of 1.6 Å or better (5σ outliers iteratively removed) are listed in Table 1. Supplementary Table S2 shows the targets observed at a resolution of 2.5 Å or better (5σ outliers iteratively removed).

Supplementary Table S2. The targets have been derived from crystallographic structures determined at a resolution of 2.5 Å or better and are listed as mean ± standard deviation. Numbers in brackets indicate the number of observations. For all targets a significant difference between means was observed across the types of $ZnCys_xHis_y$ complexes [one-way ANOVA with a Welch correction for non-homogeneity (Welch, 1951); Zn—S^γ distance: ($F_{(3,\ 217.8)}$ = 290.2, $p << 10^{-8}$); S^γ—Zn—S^γ angle: $F_{(2,\ 1042.5)}$ = 784.8, $p << 10^{-8}$; Zn—N distance: $F_{(3,\ 180.1)}$ = 36.5, $p << 10^{-8}$; N—Zn—N angle: $F_{(2,\ 180.1)}$ = 19.6, $p = 2.0 \times 10^{-8}$].

**Zn—S^γ (Å)**	**S^γ—Zn—S^γ (°)**	**Zn—N (Å)**	**N—Zn—N (°)**	**$ZnCys_xHis_y$**
2.328 ± 0.030 (10177)	109.42 ± 5.16 (15285)	n/a	n/a	Cys_4
2.316 ± 0.028 (5466)	111.83 ± 4.26 (5468)	2.055 ± 0.066 (1817)	n/a	Cys_3His_1
2.308 ± 0.033 (782)	115.40 ± 4.85 (391)	2.038 ± 0.074 (777)	105.13 ± 6.43 (391)	Cys_2His_2
2.309 ± 0.042 (47)	n/a	2.019 ± 0.072 (141)	107.51 ± 4.90 (141)	Cys_1His_3
n/a	n/a	1.983 ± 0.063 (48)	109.41 ± 6.21 (72)	His_4

S2. Geometry of $ZnCys_xHis_y$ sites

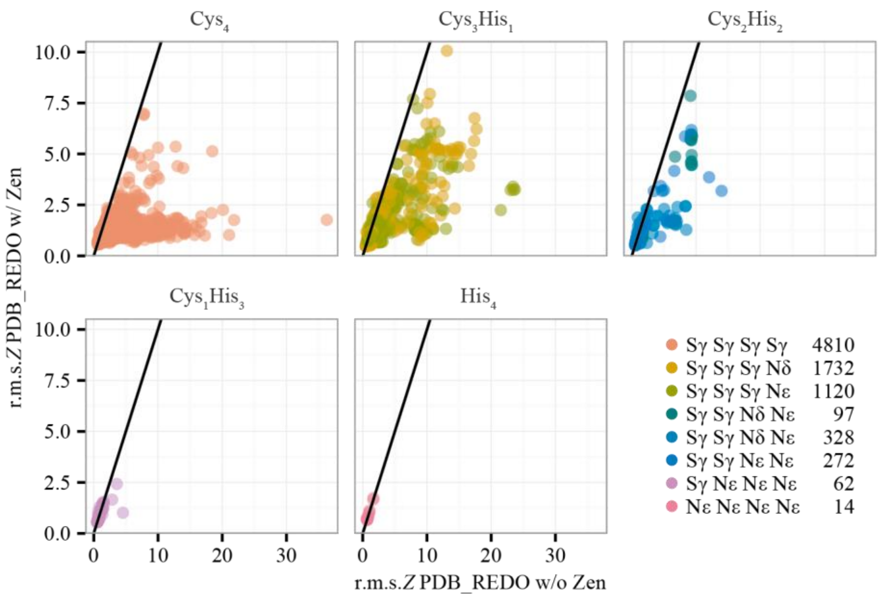

Supplementary Figure S1. r.m.s.*Z* for the five possible $ZnCys_xHis_y$ site types after *PDB_REDO* re-refinement with and without *Zen* remediation. PDB and *Zen*-remediated sites are compared in Fig. 1 in the main text.

Supplementary Figure S2. (next page). Box-and-whisker plots of the *Z* scores characterizing $ZnCys_xHis_y$ complexes in original PDB (red), *PDB_REDO* without *Zen* remediation (green) and *PDB_REDO* with *Zen* remediation (blue) structure models. The whiskers extend to the nearest value that is within 1.5 times the inter-quartile range; outliers are marked as dots. The *Z* score for 'Zn position' indicates the deviation from the expected Zn position in the tetrahedron. 4256 outliers with a *Z* score smaller than -15 or larger than 15 are not shown for clarity. 2735 of these outliers are from PDB structure models, 1440 are from *PDB_REDO* without *Zen*, and 81 are from *PDB_REDO* with *Zen* remediation, respectively.

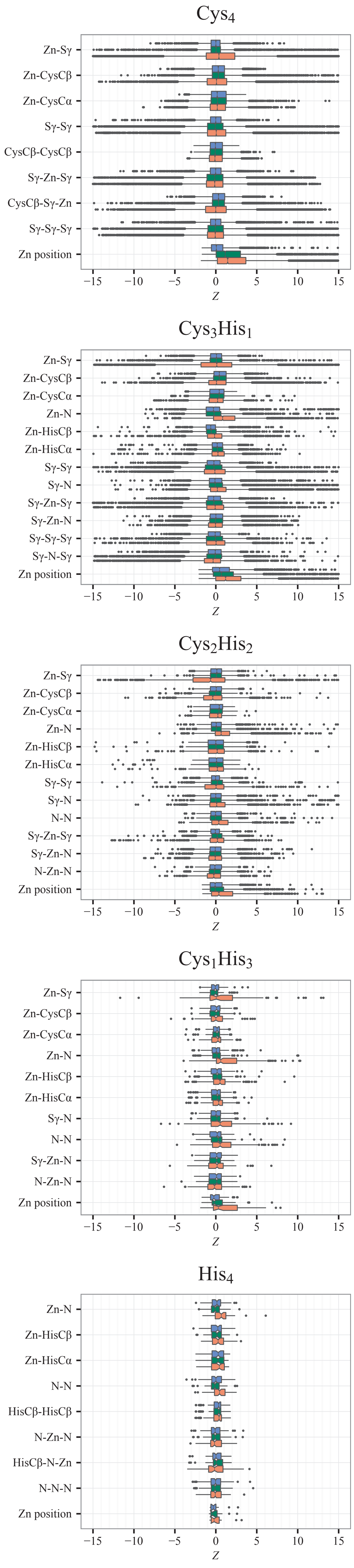

Cys4
Zn-Sγ
Zn-CysCβ
Zn-CysCα
Sγ-Sγ
CysCβ-CysCβ
Sγ-Zn-Sγ
CysCβ-Sγ-Zn
Sγ-Sγ-Sγ
Zn position
−15 −10 −5 0 5 10 15
Z
Cys3His1
Zn-Sγ
Zn-CysCβ
Zn-CysCα
Zn-N
Zn-HisCβ
Zn-HisCα
Sγ-Sγ
Sγ-N
Sγ-Zn-Sγ
Sγ-Zn-N
Sγ-Sγ-Sγ
Sγ-N-Sγ
Zn position
−15 −10 −5 0 5 10 15
Z
Cys2His2
Zn-Sγ
Zn-CysCβ
Zn-CysCα
Zn-N
Zn-HisCβ
Zn-HisCα
Sγ-Sγ
Sγ-N
N-N
Sγ-Zn-Sγ
Sγ-Zn-N
N-Zn-N
Zn position
−15 −10 −5 0 5 10 15
Z
Cys1His3
Zn-Sγ
Zn-CysCβ
Zn-CysCα
Zn-N
Zn-HisCβ
Zn-HisCα
Sγ-N
N-N
Sγ-Zn-N
N-Zn-N
Zn position
−15 −10 −5 0 5 10 15
Z
His4
Zn-N
Zn-HisCβ
Zn-HisCα
N-N
HisCβ-HisCβ
N-Zn-N
HisCβ-N-Zn
N-N-N
Zn position
−15 −10 −5 0 5 10 15
Z

S3. Zn *B* factors

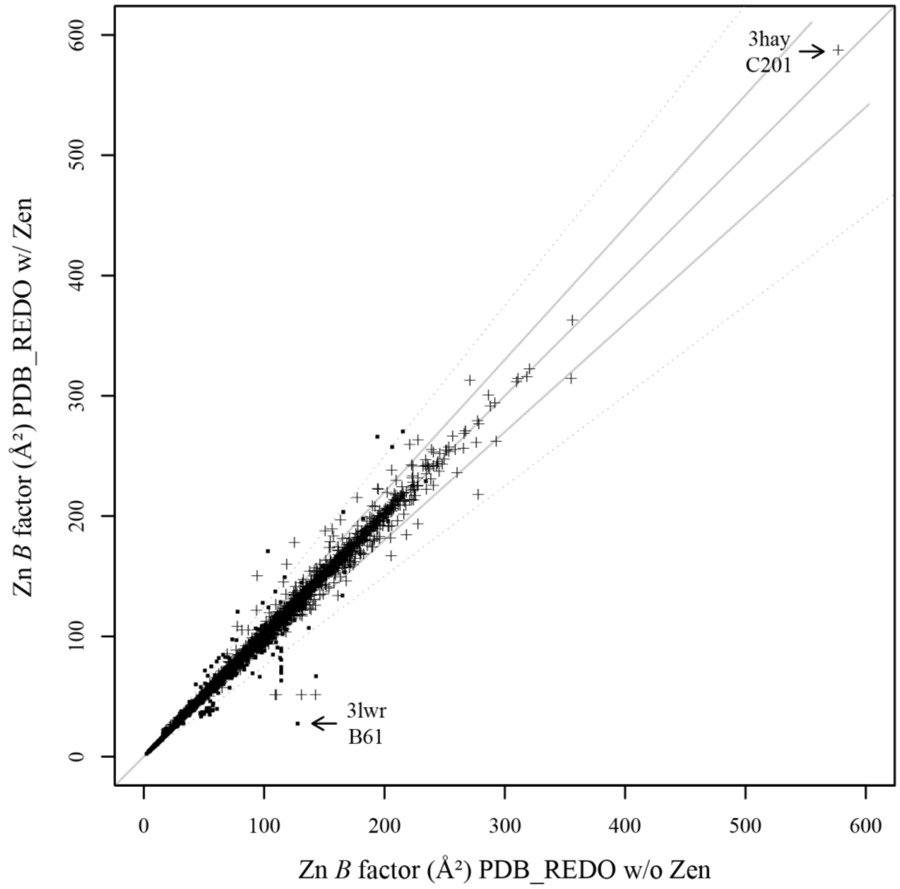

(*a*)

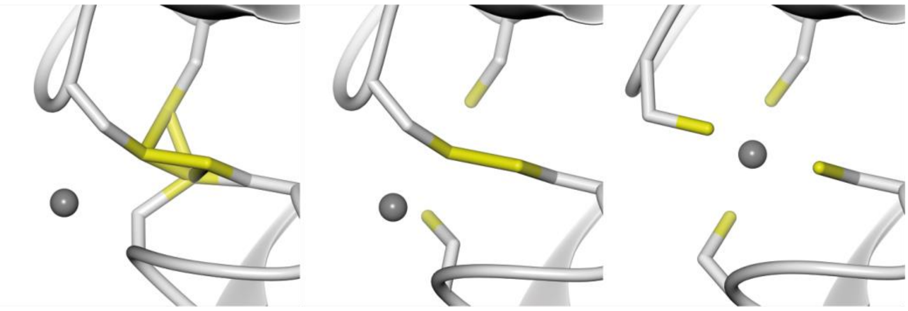

(*b*) (*c*) (*d*)

Supplementary Figure S3. (*a*) Zn *B* factors of 8305 *PDB_REDO* $ZnCys_xHis_y$ sites refined with and without *Zen* remediation with the same *B*-factor model. Plus signs indicate entries solved at a resolution worse than 3 Å. Full isotropic *B* factors are shown when the site was part of a TLS group. Entries that displayed signs of TLS-refinement artefacts have been omitted. The black diagonal line indicates identical *B* factors. The solid and dashed grey lines indicate that *B* factors with *Zen* remediation differ 10% and 25%, respectively. *Zen* remediation does not change the *B* factors significantly (two-sided paired Wilcoxon signed-rank test; V=9.9 x 10^6; *p*-value = 0.39). The *B* factors of Zn201, chain C, of H/ACA RNP [PDB 3hay; 4.99 Å (Duan *et al.*, 2009)] went up from 250 Å^2 to ~580 Å^2 high as a result of re-refinement in *PDB_REDO*. This may be due to the choice of *B*-factor model (individual *B* factors with tight restraints + TLS) in these PDB_REDO jobs. The *B* factor of the homology-related Zn61, chain B, of H/ACA RNP [PDB 3lwr; 2.2 Å (Zhou *et al.*, 2010)] is 236.7 Å^2 in the PDB entry (*b*) and 127.9 Å^2 in the *PDB_REDO* entry created without *Zen* (*c*). After *Zen* remediation (*d*) Zn61 is at the centre of the Cys_4 site and its *B* factor is 27.5 Å^2.

S4. Remaining challenges

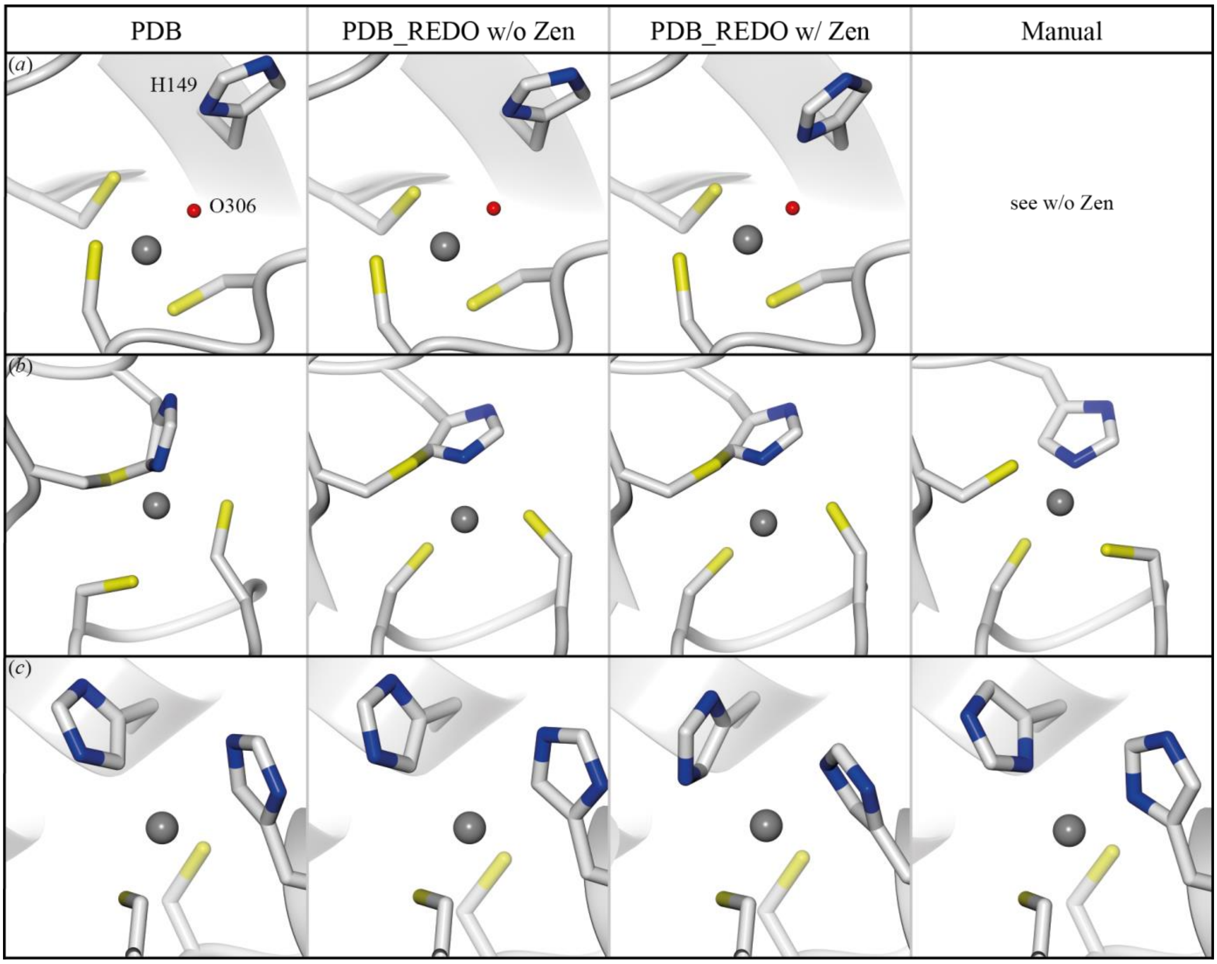

Supplementary Figure S4. (*a*) *Zen* generates distance restraints between Zn201 and His149 rather than between Zn201 and water O306 in chain A of the Hepatitis C Virus Protease [PDB 4k8b; 2.8 Å (LaPlante *et al.*, 2014)]. (*b*) Zn601, chain B, of the SARS virus nsp14-nsp10 complex [PDB 5c8s; 3.33 Å (Ma *et al.*, 2015)]. LINK records between Zn ligands prevent correct refinement. *Zen* has been adapted to deal with this, fortunately rare, type of problem. (*c*) Undetected side-chain flips of the Histidines that coordinate Zn504, chain A, of the SAGA Deubiquinating Module [PDB 4fip; 2.69 Å (Samara *et al.*, 2012)].

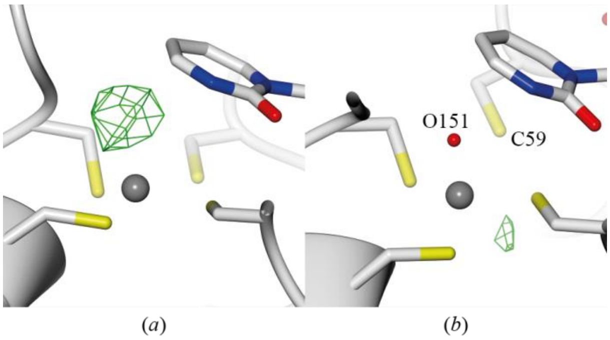

Supplementary Figure S4. Zn147 in the active site of cytidine deaminase [PDB 1mq0; 2.4 Å (Chung *et al.*, 2005)]. (*a*) In chain A the mF_o-DF_c difference density map from *PDB_REDO* (contoured at +3σ, green, and -3σ, red) within 3 Å of Zn147 indicates that the Zn-coordinating water molecule is missing. (*b*) The water molecule (O151) has been modelled in chain B. *PDB_REDO* is correct in not generating restraints between Cys59 and Zn147, but is not yet capable of adding the water in chain A.

Supplementary References

Alberts, I. L., Nadassy, K. & Wodak, S. J. (1998). *Protein Sci.* **7**, 1700–1716.

Chung, S. J., Fromme, J. C. & Verdine, G. L. (2005). *J. Med. Chem.* **48**, 658–660.

Duan, J., Li, L., Lu, J., Wang, W. & Ye, K. (2009). *Mol. Cell.* **34**, 427–439.

Harding, M. M. (2006). *Acta Cryst.* D**62**, 678–682.

Laitaoja, M., Valjakka, J. & Jänis, J. (2013). *Inorg. Chem.* **52**, 10983–10991.

LaPlante, S. R., Nar, H., Lemke, C. T., Jakalian, A., Aubry, N. & Kawai, S. H. (2014). *J. Med. Chem.* **57**, 1777–1789.

Lee, Y. M. & Lim, C. (2008). *J. Mol. Biol.* **379**, 545–553.

Ma, Y., Wu, L., Shaw, N., Gao, Y., Wang, J., Sun, Y., Lou, Z., Yan, L., Zhang, R. & Rao, Z. (2015). *Proc. Nat. Acad. Sci.* **112**, 9436–9441.

Samara, N. L., Ringel, A. E. & Wolberger, C. (2012). *Structure*, **20**, 1414–1424.

Simonson, T. & Calimet, N. (2002). *Prot. Struct. Funct. Genet.* **49**, 37–48.

Tamames, B., Sousa, S. F., Tamames, J., Fernandes, P. A. & Ramos, M. J. (2007). *Prot. Struct. Funct. Bioinf.* **69**, 466–475.

Welch, B. L. (1951). *Biometrika*, **38**, 330–336.

Zhou, J., Liang, B. & Li, H. (2010). *Biochemistry,* **49**, 6276–6281.
